# Incidental colon adenocarcinoma in a patient with an Exophytic breast mass

**DOI:** 10.1093/omcr/omag052

**Published:** 2026-05-10

**Authors:** Thomas J Sorenson, Nihkil Agrawal

**Affiliations:** Hansjorg Wyss Department of Plastic Surgery, NYU-Langone Health, 222 East 41st Street, New York, NY, 10016, United States; Hansjorg Wyss Department of Plastic Surgery, NYU-Langone Health, 222 East 41st Street, New York, NY, 10016, United States

**Keywords:** oncology, radiology, nuclear medicine, and medical imaging, radiology

**Figure 1:**
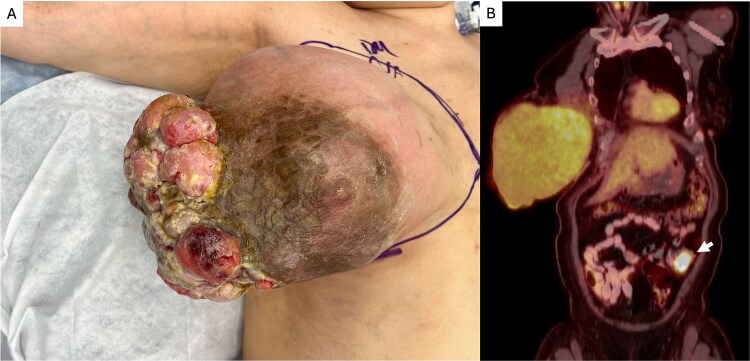
(A) Exophytic right breast mass determined to be malignant phyllodes tumor (B) PET scan demonstrating increased uptake in the descending colon ultimately determined to be an early primary malignancy of the colon.

A 59-year-old female patient presented to emergency department with 18-month history of a growing exophytic breast mass (Fig. 1). Patient was otherwise healthy and college-educated but had not seen a doctor for two decades. Biopsy determined the lesion to be malignant phyllodes tumor of the breast. Exophytic phyllodes tumors of the breast represent an uncommon and particularly aggressive form, characterized by rapid outward growth through the skin, often resulting in ulceration, and bleeding, as seen in this patient [1]. While phyllodes tumors are rare fibroepithelial neoplasms accounting for less than 1% of all breast tumors, exophytic growth reflects extreme tumor expansion and not a distinct histopathologic subtype [2, 3]. These lesions can reach substantial size over a short period of time, causing significant local morbidity and posing diagnostic and reconstructive challenges. Management is frequently complicated by compromised overlying skin, distorted breast anatomy, and the need for wide local excision or mastectomy to achieve negative margins before reconstruction and radiation [4, 5]. Our patient was taken to the operating room for radical mastectomy. After margins were cleared, the patient underwent local flap coverage of the chest wound.

As part of her workup and routine cancer screening, the patient underwent PET scan and colonoscopy, which demonstrated no sites of distant metastasis but an additional mass in the descending colon that was determined to be early primary colon adenocarcinoma. As part of their work-up in non-life-threatening situations, patients must undergo total body assessment and additional cancer screenings as indicated, which may reveal additional primary malignancies as seen in this patient. Following flap coverage of the chest wound, the patient underwent curative left hemicolectomy, and at one-year follow-up, patient remained healthy and disease-free.
