# Correction to: Incidental colon adenocarcinoma in a patient with an Exophytic breast mass

**DOI:** 10.1093/omcr/omag120

**Published:** 2026-06-16

This is a correction to: Thomas J Sorenson, Nikhil Agrawal, Incidental colon adenocarcinoma in a patient with an Exophytic breast mass, *Oxford Medical Case Reports*, Volume 2026, Issue 5, May 2026, omag052, https://doi.org/10.1093/omcr/omag052

The first name of the second co-author has been corrected to read: “Nikhil Agrawal”.

